# Structural informatic study of determined and AlphaFold2 predicted molecular structures of 13 human solute carrier transporters and their water-soluble QTY variants

**DOI:** 10.1038/s41598-022-23764-y

**Published:** 2022-11-22

**Authors:** Eva Smorodina, Igor Diankin, Fei Tao, Rui Qing, Steve Yang, Shuguang Zhang

**Affiliations:** 1 Laboratory for Computational and Systems Immunology, Department of Immunology, University of Oslo, Oslo University Hospital, Oslo, Norway; 2 Department of Computer Science, American University of Armenia, Yerevan, Armenia; 3 Laboratory of Food Microbial Technology, State Key Laboratory of Microbial Metabolism, School of Life Sciences and Biotechnology, Shanghai Jiaotong University, Shanghai, 200240 China; 4 PT Metiska Farma, Daerah Khusus Ibukota, Jakarta, 12220 Indonesia; 5 Laboratory of Molecular Architecture, Media Lab, Massachusetts Institute of Technology, 77 Massachusetts Avenue, Cambridge, MA 02139 USA

**Keywords:** Computational models, Protein folding, Protein function predictions, Protein structure predictions, Biological techniques, Computational biology and bioinformatics

## Abstract

Solute carrier transporters are integral membrane proteins, and are important for diverse cellular nutrient transports, metabolism, energy demand, and other vital biological activities. They have recently been implicated in pancreatic cancer and other cancer metastasis, angiogenesis, programmed cell death and proliferation, cell metabolism and chemo-sensitivity. Here we report the study of 13 human solute carrier membrane transporters using the highly accurate AlphaFold2 predictions of 3D protein structures. In the native structures, there are hydrophobic amino acids leucine (L), isoleucine (I), valine (V) and phenylalanine (F) in the transmembrane alpha-helices. These hydrophobic amino acids L, I, V, F are systematically replaced by hydrophilic amino acids glutamine (Q), threonine (T) and tyrosine (Y), thus the QTY code. Therefore, these QTY variant transporters become water-soluble without requiring detergents. We present the superposed structures of these native solute carrier transporters and their water-soluble QTY variants. The superposed structures show remarkable similarity with RMSD ~ 1 Å–< 3 Å despite > 46% protein sequence substitutions in transmembrane alpha-helices. We also show the differences of surface hydrophobicity between the native solute carrier transporters and their QTY variants. Our study may further stimulate designs of water-soluble transmembrane proteins and other aggregated proteins for drug discovery and biotechnological applications.

## Introduction

It is well-known that pancreatic cancer is a highly fatal disease^1–3^. Many active basic and clinical research efforts have been made, but no effective treatment is yet in sight. Recently, several solute carrier (SLCs) transporters have come to light that are involved in six crucial routes of pancreatic cancer resulting in very poor prognoses including: (i) cancer cell proliferation, (ii) programmed cell death, (iii) invasion and metastasis, (iv) angiogenesis, (v) cellular metabolism and (vi) chemo-sensitivity. These SLC transporters move a broad range of key substrates including (a) amino acids, (b) nucleotides, (c) essential metal ions including zinc, magnesium, sodium, lithium, copper, other organic ions, NH_4_^+^, HCO_3_^−^, (d) short chain fatty acids, (e) co-factors, vitamins, and (f) other organic ions and compounds into cells. Some of these SLCs are involved in pancreatic cancer metastasis and angiogenesis, others are involved in proliferation and programmed cell death, as well as cellular metabolism and chemo-sensitivity (Table 1)^1–3^. With these new insights, these SLCs not only should be the focus of intense research, but should also be the targets for new therapeutic strategies to treat pancreatic cancer and perhaps other cancer metastasis^1–4^.

**Table 1:** The solute carrier transporters relevant in pancreatic cancer.

Transporter	Cancer characteristics	Transporter substrates
1) SLC2A1	Metastasis/Cellular metabolism	Glucose and other sugars
2) SLC7A11	Metastasis/Cell death/Proliferation/Chemo-sensitivity	Cysteine, Glutamate
3) SLC4A4	Metastasis/Angiogenesis	Na^+^, HCO^−^
4) SLC1A5	Proliferation/Cellular metabolism/Chemo-sensitivity	Ala, Ser, Cys, Thr, Asn, Gln
5) SLC7A5	Proliferation/ Cellular metabolism	Large AA, DOPA, BCH
6) SLC29A1	Chemo-sensitivity	Nucleotides
7) SLC39A6	Metastasis/Proliferation	Zn^2+^
8) SLC39A3	Proliferation	Zn^2+^
9) SLC9A1	Metastasis	Na^+^, Li^+^, NH_4_^+^, H^+^,
10) SLC6A14	Programmed cell death	Neutral and cationic AA
11) SLC4A7	Proliferation	Na^+^, HCO^−^
12) SLC5A8	Proliferation	Short chain fatty acids
13) SLC41A1	Proliferation	Mg^2+^

The Table 1 is summarized from Wu et al. (Ref^1^). For details, please consult the original review.

There are 46 distinct gene families of SLC transporters comprising 384 genes in the human genome^2,5^. Some members of these SLC transporters have recently been found to be involved in several key aspects of cancer including fatal pancreatic cancer (Table 1). One of the key characteristics of cancer is deregulation of cellular energetics, namely the insatiable energy demand including sugars and nutrients through upregulating the transporters^1,3,6^. By effectively blocking the key logistics of transport systems to the cancer cells, we may be able to add another tool to more effectively treat caners.

SLC transporters are involved in multiple cellular pathways for pancreatic cancer and others in a single pathway. For example, several are involved in metastasis, programmed cell death, proliferation, chemo-sensitivity (Table 1) including SLC2A1 (also called GLUT1)^7–9^, SLC7A11^10^, SLC4A4^11^, SLC1A5^12^ and SLC7A5^13,14^, SLC39A6^15,16^. On the other hand, others are only involved a single pathway (Table 1) including SLC39A3^17–19^, SLC9A1^20–22^, SLC6A14^23^, SLC4A7^24^, SLC5A8^25,26^ and SLC41A1^27^. The motivation of this study is to endeavor to discover new drugs and to generate therapeutic monoclonal antibodies, by targeting those that are involved in multiple pathways.

**Figure 1:**
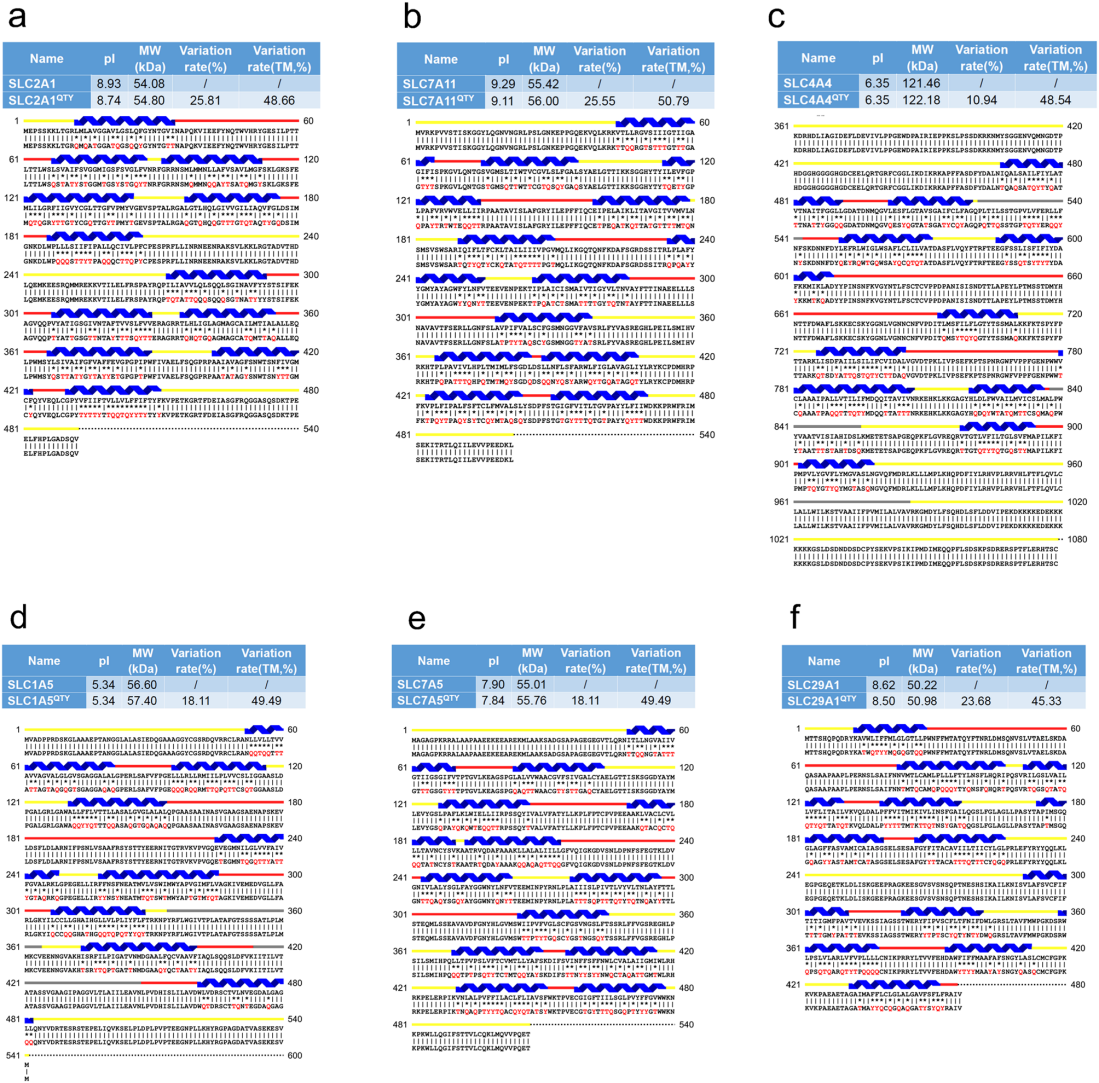
Protein alignments of six solute carrier transporters with their QTY variants. The symbols | and * indicate the identical and different amino acids, respectively. Please note the Q, T and Y amino acid replacement (red). The alpha-helices (blue) are shown above the protein sequences. The loop color codes are: internal (yellow) and external (red). Features of natural and QTY variants are: molecular weight, pI, total variation % and transmembrane variation %. The alignments are: (**a**) SLC2A1 versus SLC2A1^QTY^, (**b**) SLC7A11 versus SLC7A11^QTY^, (**c**) SLC4A4 versus SLC4A4^QTY^, (**d**) SLC1A5 versus SLC1A5^QTY^, (**e**) SLC7A5 versus SLC7A5^QTY^, (**f**) SLC29A1 versus SLC29A1^QTY^. Although there are significant QTY changes for the TM alpha-helix changes, > 45% for SLC29A1, > 50% for SLC7A11, their changes of molecular weight and pI are insignificant (Table 2).

**Figure 2:**
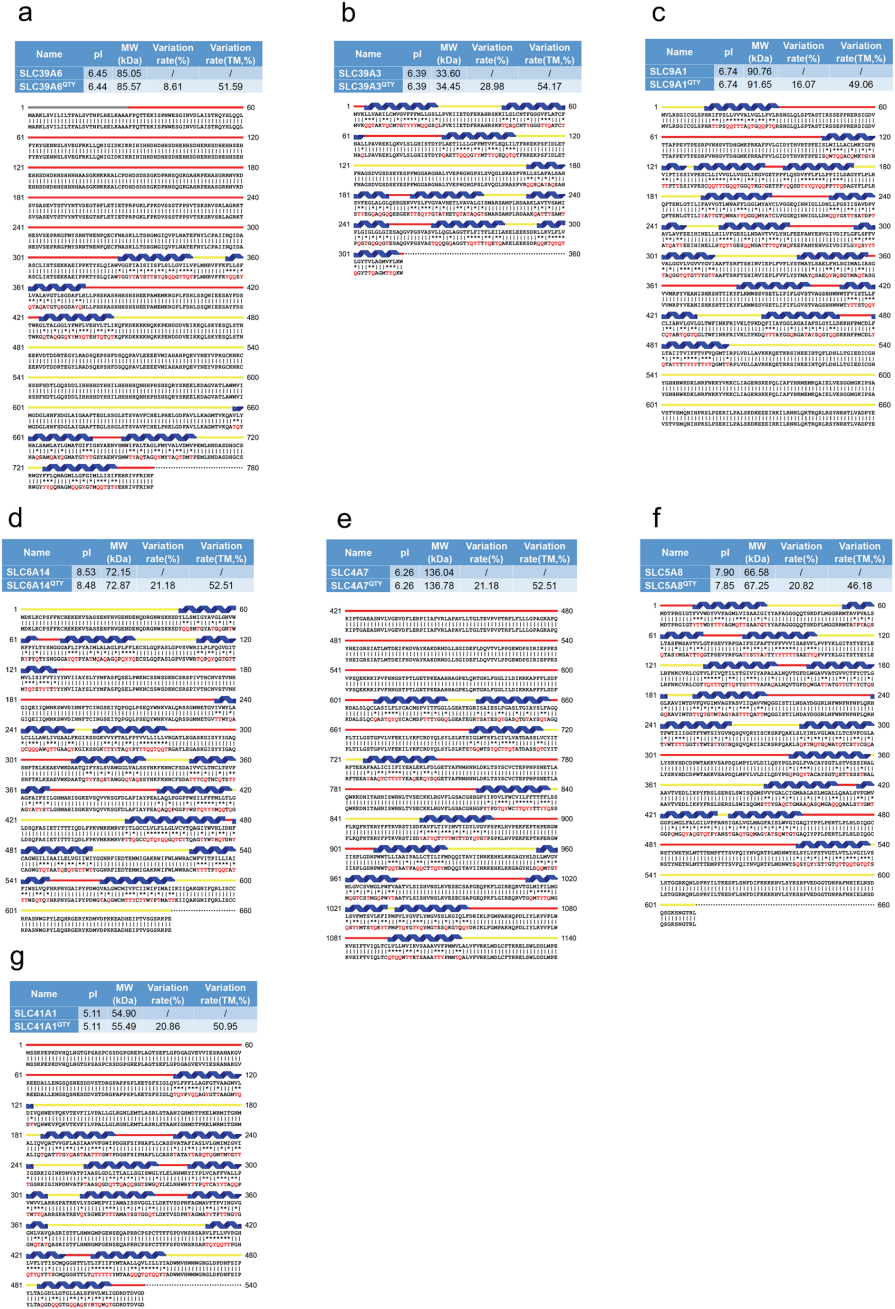
Protein alignment of eight native solute carrier transporters with their water-soluble QTY variants. All characteristics and color codes are referred in Fig. 1. The alignments are: (**a**) SLC39A6 versus SLC39A6^QTY^, (**b**) SLC39A3 versus SLC39A3^QTY^, (**c**) SLC9A1 versus SLC9A1^QTY^, (**d**) SLC6A14 versus SLC6A14^QTY^, (**e**) SLC4A7 versus SLC4A7^QTY^, (**f**) SLC5A8 versus SLC5A8^QTY^, (**g**) SLC41A1 versus SLC41A1^QTY^ (see Tables 2, 3).

SLC transporters have multiple transmembrane (TM) helices. Most have 10TM-12TM helices depending on their transporting substrates with the longest having 13TM (SLC5A8 for short chain fatty acids). Several of these SLC transporters have large N-termini and extracellular loops (Figs. 1, 2) of ~ 30–60 amino acids. Those with large extracellular loops are more likely to be better targets for generating therapeutic monoclonal antibodies since these extracellular loops may stimulate robust immune responses during immunizations.

On 15 July 2021, Google DeepMind announced the AlphaFold2, and at the same time David Baker’s lab introduced RoseTTAFold as machine learning revolutionary tool for the very accurate prediction of protein structures^28–30^. AlphaFold2 and, to a lesser extent, RoseTTAFold, have already made an enormous impact on our understanding of 350,000 protein structures. On July 28, 2022, DeepMind released 214 million protein structures, nearly all known protein structures. AlphaFold2 predicts the structures with very high accuracy for 35% of all protein structures (~ 75 million), and with high confidence for another 45% (~ 96.3 million). AlphaFold2 has truly started a new era of digital biology. Nevertheless, academic investigators, the pharmaceutical and biotech companies must still ultimately study the physical structures of proteins, including SLC membrane transporters since the structures are vital to understanding how substrates including sugars, amino acids, essential ions, organic molecules, drugs, or other essential nutrients are transported across highly regulated and controlled cell membranes.

We previously applied the QTY (Glutamine, Threonine, Tyrosine) code to design several detergent-free transmembrane (TM) protein chemokine receptors and cytokine receptors for various uses using conventional computing programs to simulate several G protein-coupled receptors. Each took ~ 5 weeks to complete the simulation^33–35^. The expressed and purified water-soluble proteins exhibited predicted characteristics and retained ligand-binding activity^31–35^. In July 2021, we prepared QTY variant protein structure predictions using AlphaFold2, achieving better results in 1–2 hours^36,37^, rather than ~ 5 weeks for each molecular simulation using GOMoDo, AMBER and YASARA programs^31–33^. We also produced a program and website for generating the membrane protein water-soluble QTY variants^38^.

Here, we report using AlphaFold2 to design water-soluble QTY variants of the 13 solute carrier transporters, and to directly compare with their counterpart native structures. In addition to targeting the key nutrients and ion uptake activity of cancer cells, these QTY variant water-soluble SLC transporters can prospectively find many additional applications. Working with water-soluble QTY variants may substantially accelerate the discovery and development of therapeutic and diagnostic biologicals.

## Results and discussions

### Protein sequence alignments and other characteristics

We aligned the native SLC transporters with their QTY variants. Despite significant QTY replacement of hydrophobic residues in the transmembrane domains (~ 44–55%) in the SLC transporters, the isoelectric focusing point pI and molecular weight remain rather similar (Figs. 1 and 2, Table 2). This is because Q, T, Y amino acids do not introduce any charges, they only introduce water-soluble side chains. Q (glutamine) side chains form 4 water hydrogen bonds, 2 donors through –NH2, and 2 acceptors through oxygen on –C=O; the sidechains –OH of T (threonine) and Y (tyrosine) form 3 water hydrogen bonds, 1 donor from H (hydrogen) and 2 acceptors from O (oxygen).

**Table 2:** Characteristics of native solute carrier transporters and their water-soluble QTY variants.

Name	RMSD	pI	MW (KD)	TM variation (%)	Overall variation (%)
SLC2A1	–	8.93	54.08	–	–
SLC2A1^QTY^	2.281 Å	8.74	54.8	48.66	25.81
SLC7A11	–	9.29	55.42	–	–
SLC7A11^QTY^	0.933 Å	9.11	56	50.79	25.55
SLC4A4	–	6.35	121.46	–	–
SLC4A4^QTY^	0.445 Å	6.35	122.18	48.54	10.94
SLC1A5	–	5.34	56.6	–	–
SLC1A5^QTY^	0.965 Å	5.34	55.01	49.49	18.11
SLC7A5	–	7.9	55.76	–	–
SLC7A5^QTY^	1.815 Å	7.84	55.63	49.49	18.11
SLC29A1	–	8.62	50.22	–	–
SLC29A1^QTY^	1.512 Å	8.5	50.98	45.33	23.68
SLC39A6	–	6.45	85.05	–	–
SLC39A6^QTY^	0.931 Å	6.44	85.57	51.59	8.61
SLC39A3	–	6.39	33.6	–	–
SLC39A3^QTY^	1.567 Å	6.39	34.45	54.17	22.98
SLC9A1	–	6.74	90.76	–	–
SLC9A1^QTY^	0.905 Å	6.74	91.65	49.06	16.07
SLC6A14	–	8.53	72.15	–	–
SLC6A14^QTY^	0.521 Å	8.48	72.87	52.51	21.18
SLC4A7	–	6.26	136.04	–	–
SLC4A7^QTY^	0.426 Å	6.26	136.78	52.51	21.18
SLC5A8	–	7.9	66.58	–	–
SLC5A8^QTY^	1.593 Å	7.85	67.25	46.18	20.82
SLC41A1	–	5.11	54.9	–	–
SLC41A1^QTY^	1.914 Å	5.11	55.49	50.95	20.86

Residue mean-square distance (RMSD) in Å, Isoelectric focusing (pI), Molecular weight (MW), Transmembrane (TM), – = not applicable. The internal and external loops have no changes, the overall changes are not insignificant, and the TM changes are rather large.

Since the electron density maps share remarkable structure similarities between leucine (L) versus glutamine (Q); isoleucine (I), valine (V) versus threonine (T); and phenylalanine (F) versus tyrosine (Y), the QTY code selects 3 neutrally polar amino acids: glutamine, threonine and tyrosine to replace 4 hydrophobic amino acids leucine, isoleucine, valine and phenylalanine. After applying the QTY code, the hydrophobic amino acids in the transmembrane segments are replaced by Q, T, and Y, therefore the transmembrane segments have significantly reduced hydrophobicity. For example, SLC39A3 and SLC29A1 differ > 54 and > 45% from their water-soluble QTY variants, respectively, in their transmembrane alpha-helical segments. (Figs. 1, 2, Table 2).

Other characteristics are also notable. The pIs (isoelectric-focusing points) vary, some in the acidic and some in the basic range. For example, native SLC7A11 has a basic pI of 9.29. On the other hand, SLC1A5 has an acidic pI of 5.34. Others including SLC9A1 have a near neutral pI of 6.74 (Table 2). It is noted that the pIs are identical for the native and QTY variants for SLC4A4 (pI 6.35), SLC39A3 (pI 6.39), SLC4A7 (pI 6.26), and SLC41A1 (pI 5.11) despite the large number of QTY substitutions. The reason is that Glutamine, Threonine, Tyrosine (Q, T, Y) have neither positive nor negative charges at neutral pH. Therefore, substitutions of Q, T, Y do not change the pIs. This is significant because altered pIs could cause non-specific interactions.

Moreover, while there are between > 45– > 54% QTY substitutions in the transmembrane helices, the molecular weights of the native and QTY variants differ by only a few hundreds of Daltons. This is due to a) the substitutions of CH_3_- on Lue and Val, by -OH groups to Gln (Q) and Thr (T), and b) the addition of OH- on Tyr (Y). These increase the protein molecular weights (Figs. 1, 2, Table 2).

### Superposition of native transporters and their water-soluble QTY variants

In our current study, the native SLC structures determined by X-ray crystal or CryoEM were superimposed and compared to their QTY variants. The molecular structures of native SLC transporters are already available for SLC2A1 (PDB: 6THA)^39^, SLC7A11 (PDB: 7PV9)^40^, SLC4A4 (PDB: 6CAA)^41^, SLC1A5 (PDB: 5LMM)^42^, SLC7A5 (PDB: 6IRS)^43^, SLC29A1 (PDB: 6OB6)^44^. The superposed structures are performed for: SLC2A1^Crystal^ versus SLC2A1^QTY^, SLC7A11^CryoEM^ versus SLC7A11^QTY^, SLC4A4^CryoEM^ versus SLC4A4^QTY^, SLC1A5^Crystal^ versus SLC1A5^QTY^, SLC7A5^CryoEM^ versus SLC7A5^QTY^ and SLC29A1^Crystal^ versus SLC29A1^QTY^ (Tables 2 and 3).

**Table 3:** RMSD between native solute carrier transporters, their water-soluble QTY variants, and crystal structures.

Name	PDB	RMSD^Native/PDB^	RMSD^QTY/PDB^
SLC2A1	6THA	2.969	1.053
SLC7A11	7P9V	2.682	1.548
SLC4A4	6CAA	1.588	1.497
SLC1A5	5LM4	0.607	1.272
SLC7A5	7DSK	2.523	1.008
SLC29A1	6OB7	0.619	1.198

Residue mean-square distance (RMSD) in Å, – = not applicable. All RMSD values are below 3 Å and show good superposition between structures. All models and scripts for structure preparation and superposition in PyMOL are presented in the GitHub repository: https://github.com/eva-smorodina/slc.

**Figure 3:**
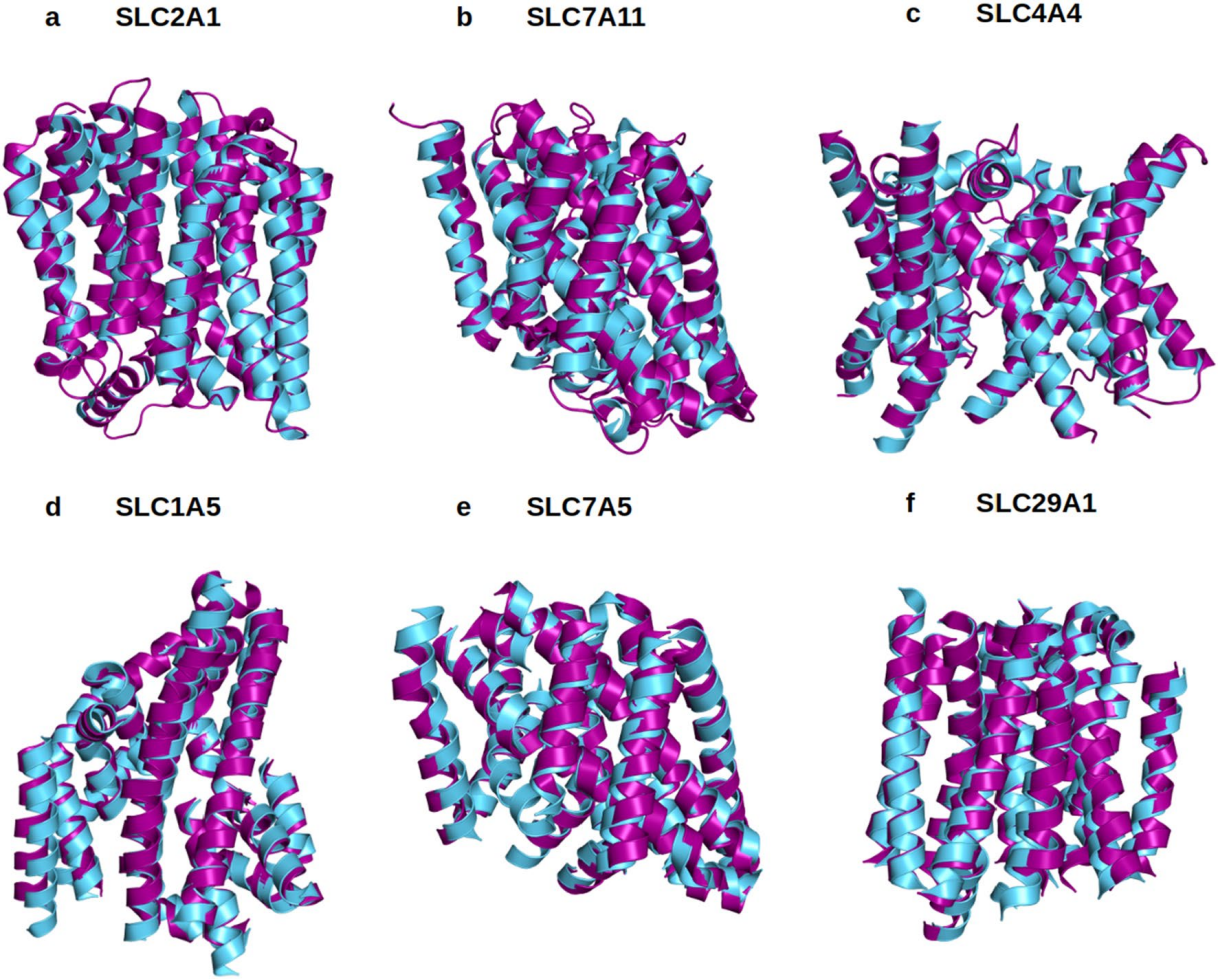
Superposed 6 solute carrier transporter crystal or CryoEM structures with QTY variants predicted by AlphaFold2. The X-ray crystal or CryoEM structures of the native transporters are obtained from the Protein Data Bank (PDB). The crystal or CryoEM structures (magenta) are superposed with QTY variants (cyan) predicted by AlphaFold2. The RMSD (Å) for each structure is in Table 2. (**a**) SLC2A1^Crystal^ versus SLC2A1^QTY^ (2.281 Å), (**b**) SLC7A11^CryoEM^ versus SLC7A11^QTY^ (0.933 Å), (**c**) SLC4A4^CryoEM^ versus SLC4A4^QTY^ (0.445 Å), (**d**) SLC1A5^Crystal^ versus SLC1A5^QTY^ (0.965 Å), (**e**) SLC7A5^CryoEM^ versus SLC7A5^QTY^ (1.815 Å), (**f**) SLC29A1^Crystal^ versus SLC29A1^QTY^ (1.512 Å). These superposed structures display that the crystal or CryoEM structures and their QTY variants have very similar molecular structures (Tables 2, 3). For clarity of direct comparisons, the N-terminus and C-terminus are deleted.

The experimentally-determined native structures and their in-silico-determined water-soluble QTY variants superposed within a few Å. Their RMSDs are as follows: SLC2A1 versus SLC2A1^QTY^ (2.281 Å); for SLC7A11 versus SLC7A11^QTY^ (0.933 Å); SLC4A4 versus SLC4A4^QTY^ (0.445 Å); SLC1A5 versus SLC1A5^QTY^(0.965 Å); SLC7A5 versus SLC7A5^QTY^ (1.815 Å); and SLC29A1 versus SLC29A1^QTY^ (1.512 Å). (Fig. 3, Table 2). It can be seen from Fig. 3, these molecular structures, experimentally-determined and AlphaFold2-predicted visibly superpose very well. These results show that despite > 45% QTY substitutions in the transmembrane alpha-helices in the water-soluble QTY variants, their structures share rather similar 3-dimentional folds. These closely superposed structures perhaps confirm that the AlphaFold2’s predictions are highly accurate, since the predicted native structures are directly superposed with the experimentally determined X-ray crystal structures. Our AlphaFold2 predicted structures show the significant structural similarity between the native SLC transporter and their water-soluble QTY variants.

**Figure 4:**
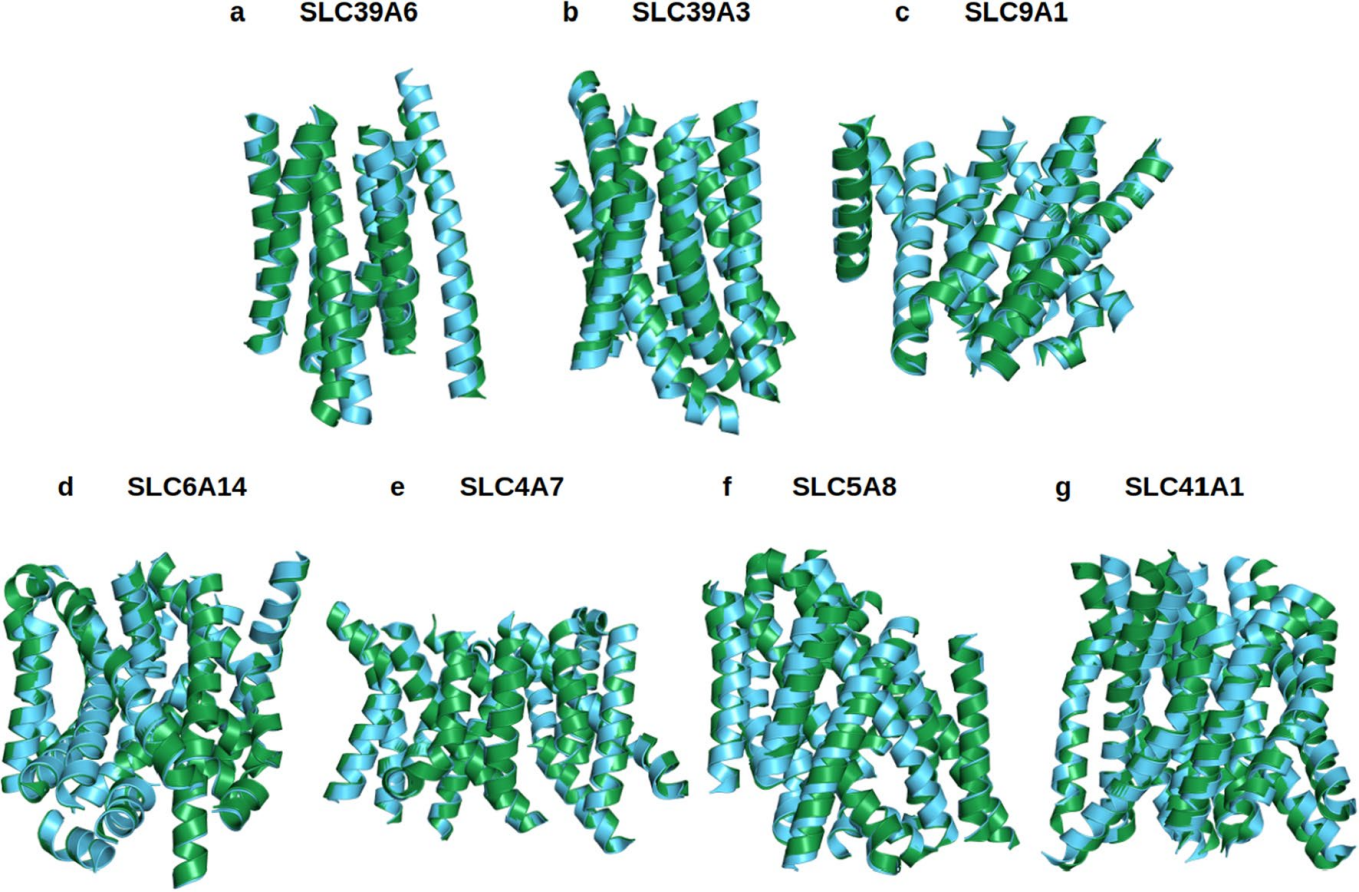
Superposed 7 native solute carrier transporters and their QTY variants that were predicted by AlphaFold2. The native structures (green) and their water-soluble QTY variants (cyan). For the superposed structures, the RMSD is in Å ( ). (**a**) SLC39A6 versus SLC39A6^QTY^ (0.931 Å) (**b**) SLC39A3 versus SLC39A3^QTY^ (1.567 Å), (**c**) SLC9A1 versus SLC9A1^QTY^ (0.905 Å), (**d**) SLC6A14 versus SLC6A14^QTY^ (0.521 Å), (**e**) SLC4A7 versus SLC4A7^QTY^ (0.426 Å), (**f**) SLC5A8 versus SLC5A8^QTY^ (1.593 Å), (**g**) SLC41A1 versus SLC41A1^QTY^ (1.914 Å). The large N- and C-termini are removed for clarity. Please see Tables 2 and 3. For clarity, N-terminus, C-terminus and large loops are deleted.

Because the X-ray crystal or CryoEM structures of the other 7 native SLCxxxx are not yet available, including SLC39A4, SLC39A6, SLC39A3, SLC9A1, SLC6A14, SLC4A7, SLC5A8 and SLC41A1, AlphaFold2 tool is used for the structural predictions. The RMSD in Å (residue mean-square distances) for these superposed structures are displayed (Fig. 4, Table 2). The examples are: SLC39A6 versus SLC39A6^QTY^ (0.931 Å); SLC39A3 versus SLC39A3^QTY^ (1.567 Å); SLC9A1 versus SLC9A1^QTY^ (0.905 Å); SLC6A14 versus SLC6A14^QTY^ (0.521 Å); SLC4A7 versus SLC4A7^QTY^ (0.426 Å); SLC5A8 versus SLC5A8^QTY^ (1.593 Å); and SLC41A1 versus SLC41A1^QTY^ (1.914 Å) (Table 2). The AlphaFold2-predicted structures of both natural SLCs and their water-soluble SLC variants superpose very well implying that they share comparable structures despite the significant substitutions in the transmembrane alpha-helices (45–54%).

### Analysis of the hydrophobic surface of native transporters and the water-soluble QTY variants

It is known that the natural SLCxxxx have high hydrophobicity content, especially in the transmembrane alpha-helical segments. They are inherently insoluble in water and require surfactants to solubilize and stabilize them. These natural transporters quickly self-associate to form unstructured aggregation, precipitation, and no longer biologically functional without the highly selected surfactants.

**Figure 5:**
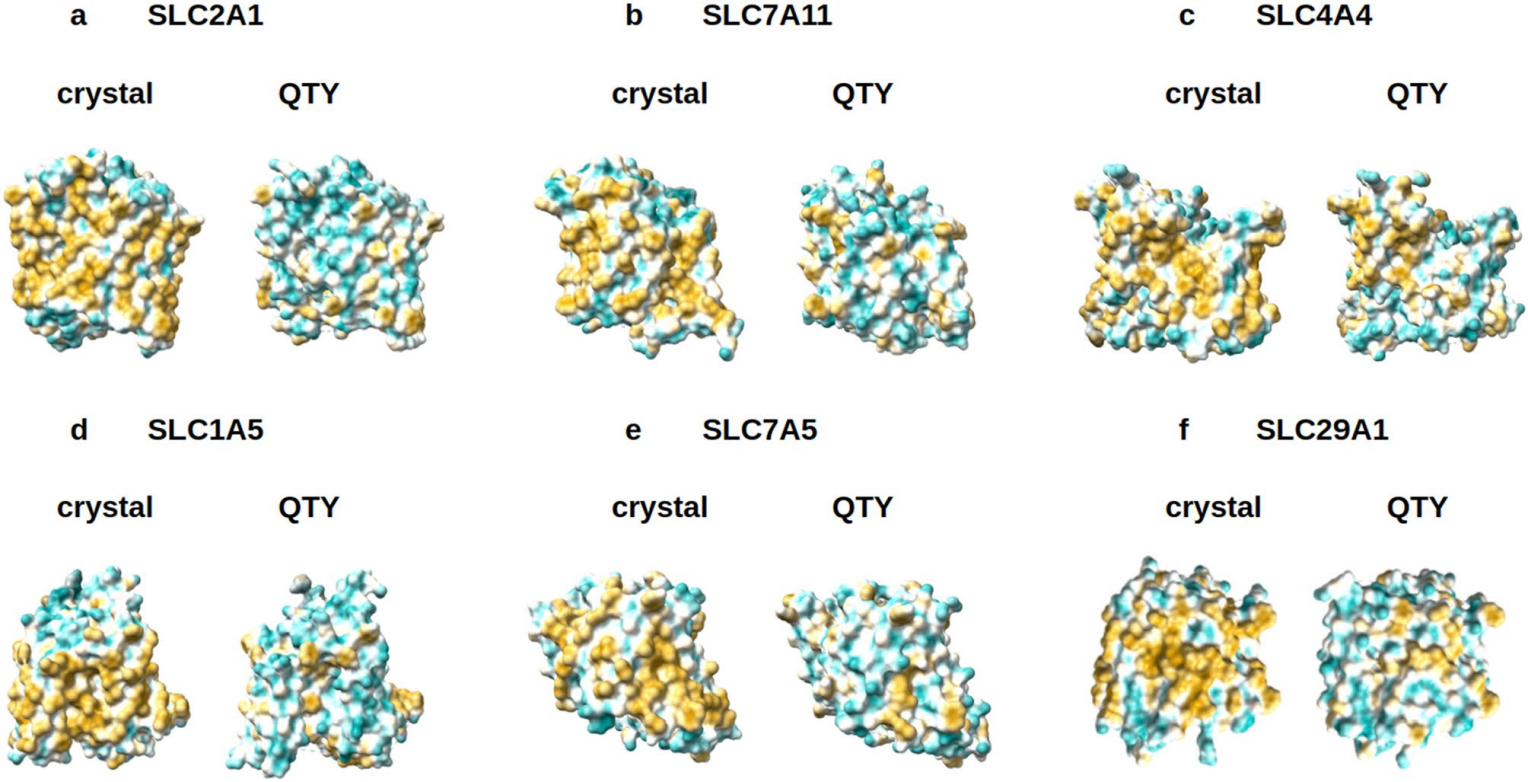
Hydrophobic surface of 6 crystal structures of solute carrier transporters and the designed QTY variants. The native solute carrier transporters have many hydrophobic residues L, I, V and F in the transmembrane helices. After Q, T, and Y replacement of the L, I, V, F, the surfaces are much more hydrophilic. The hydrophobic surface (brownish) of the native transporters become more cyan color: (**a**) SLC2A1 versus SLC2A1^QTY^, (**b**) SLC7A11 versus SLC7A11^QTY^, (**c**) SLC4A4 versus SLC4A4^QTY^, (**d**) SLC1A5 versus SLC1A5^QTY^, (**e**) SLC7A5 versus SLC7A5^QTY^, (**f**) SLC29A1 versus SLC29A1^QTY^. The hydrophobic surface is largely reduced on the transmembrane helices for the QTY variants. These QTY variants converted to water-soluble form. For clarity, the N-terminus and C-terminus are deleted.

**Figure 6:**
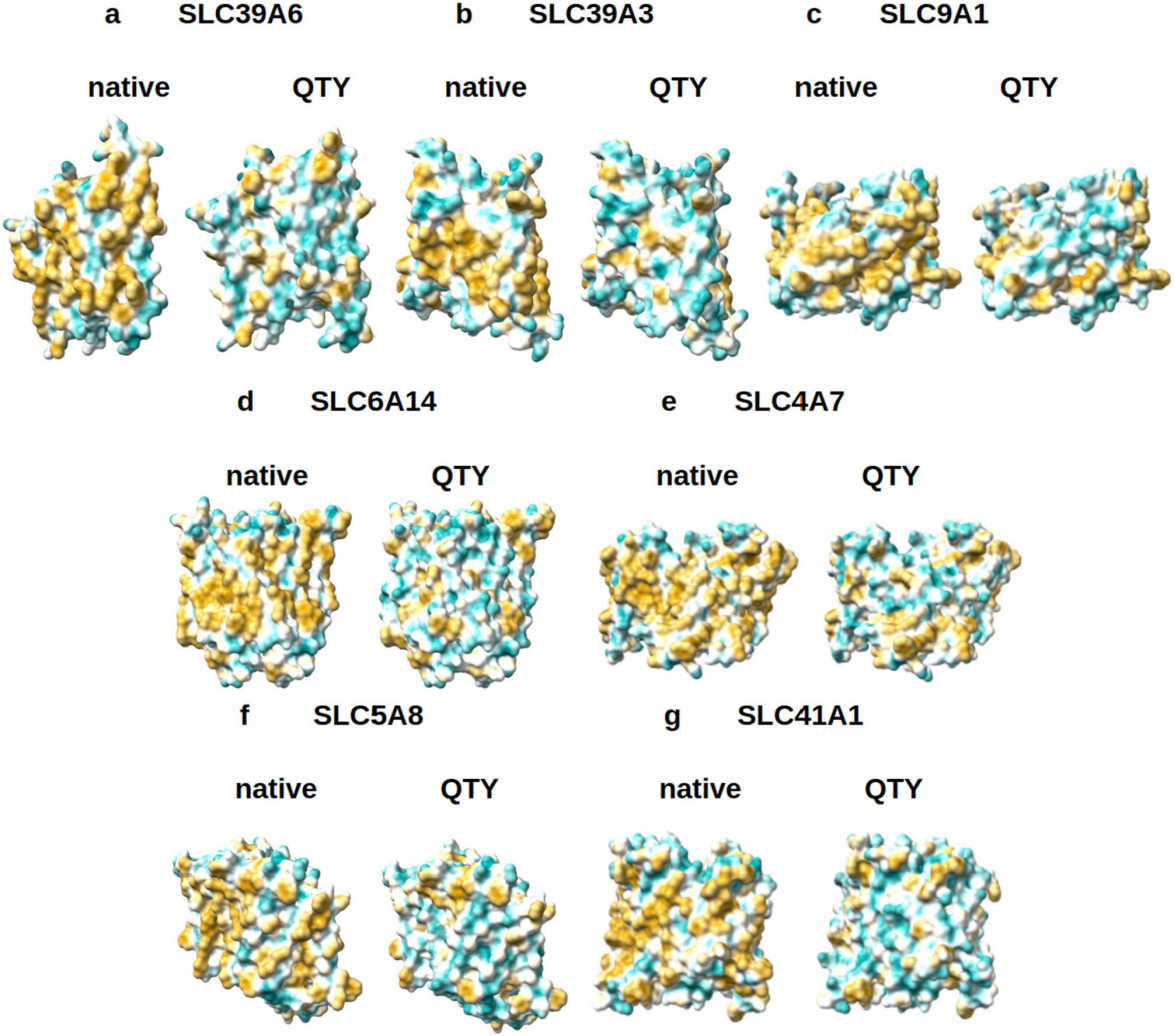
Hydrophobic surface of 8 AlphaFold2 predicted native solute carrier transporters and their QTY variants. The pairwise of AlphaFold2 predicted native transporters with hydrophobic surface (brownish color), and the QTY variant transporters (cyan color). (**a**) SLC39A6 versus SLC39A6^QTY^, (**b**) SLC39A3 versus SLC39A3^QTY^, (**c**) SLC9A1 versus SLC9A1^QTY^, (**d**) SLC6A14 versus SLC6A14^QTY^, (**e**) SLC4A7 versus SLC4A7^QTY^, (**f**) SLC5A8 versus SLC5A8^QTY^, (**g**) SLC41A1 versus SLC41A1^QTY^. For clarity, the large N- terminus and C-terminus are deleted.

In the natural SLCxxxx, the 6TM-12TM alpha-helices are directly embedded in the hydrophobic lipid bilayer. The hydrophobic side chains of phenylalanine, isoleucine, leucine, and valine interact with the hydrophobic lipid bilayers. Thus, the 6TM-12TM alpha-helices exhibit water-repelling hydrophobic surfaces (Figs. 5, 6).

After QTY substitutions of hydrophobic amino acids L, I, V, F, with hydrophilic amino acids Q, T and Y, the hydrophobic surfaces are decreased (Figs. 5, 6). The QTY changes hydrophobic 6TM-12TM into hydrophilic 6TM-12TM, without, however, significantly changing the alpha-helical molecular structures, as shown in Fig. 3. Analogous reproducible experimental results reported for chemokine and cytokine receptors in our previous publications^31–35^. However, our experimental results showed that the structure integrity, stability, and ligand-binding activities have been retained from the water-soluble QTY-variant chemokine receptors and cytokine receptors^31–35^.

There are three chemically distinct alpha-helix types. Type I: the water-soluble hydrophilic alpha-helix, commonly water-soluble enzymes in the cellular cytosols and extracellular circulating proteins including antibodies, protein and peptide hormones and more; Type II: the water-insoluble hydrophobic alpha-helix commonly in transmembrane proteins including hormone receptors, transporters, ion channels, G protein-coupled receptors, photosynthesis systems; and Type III: the amphiphilic alpha-helix, like a Janus that have a hydrophobic face on one side and a hydrophilic face on the other side. These three chemically distinct alpha-helical types have very similar structures, regardless their hydrophobicity and hydrophilicity^45–49^. This is the molecular foundation of the QTY code.

### AlphaFold2 predictions

For over 65 years, scientists have made great efforts to predict protein folding. It was the dream of structural biologists and protein scientists to predict protein folding rapidly and accurately. With the advent of AlphaFold2 through machine learning, the tool is now available to predict protein structure by all scientists, almost free of charge. We can now study previously unattainable protein structures, particularly membrane-embedded transmembrane proteins.

Systematic bioinformatic studies revealed that in most organisms, ~ 20–30% genes code for membrane proteins^50^. It is known that the human genome codes for ~ 24% membrane proteins^51^. But structural determination of a single transmembrane protein is an extremely difficult process, traditionally requiring even decades of endeavor. There are many barriers, from gene expression, protein production, detergent selection, purification, detergent exchange, to maintaining their long-term stability and integrity as well as maintaining functionality to avoid irreversible aggregation. The numbers of integral transmembrane protein structures lag far behind water-soluble proteins. Recently, several groups have systematically analyzed the known structures and at least 16 different folds of ~ 400 members, 65 families of the solute carrier transporter^52,53^. These studies further provide insights into molecular structures and functions of these transporters.

Applying AlphaFold2 accurate protein structure predictions, we can directly compare the native structure with an AlphaFold2 predicted water-soluble QTY variant. With the QTY variant, expressed in various cells, it becomes possible to overcome the high barriers of studying membrane embedded transmembrane proteins.

We previously reported^36,37^ use of the AlphaFold2 tool to predict structures of the water-soluble variants of G protein coupled receptor chemokine receptors and glucose transporters, and compared them to the known experimentally-determined crystal or CryoEM structures.

One of the questions concerns what would be the utility of these water-soluble transporters that can no longer transport molecules cross the membrane since the water-soluble QTY variants can no longer insert themselves into the lipid bilayer membrane. It is plausible that after these transporters are rendered water-soluble, they can be used: (1) as water-soluble antigens to generate useful monoclonal antibodies in animals since they have many extracellular loops including few large loops, and (2) these anti-SLCxxx antibodies could be useful as research reagents for an assay system to study the transporters in tissue cell cultures and in vivo. These specific monoclonal antibodies can perhaps also be useful (a) as therapeutics to treat diseases including pancreatic cancer, (b) as diagnostic reagent to monitor cancer treatments and perhaps (c) for early pancreatic cancer detection.

Our current study using the AlphaFold 2 demonstrates that the water-soluble QTY-variant structures of SLC transporters are substantially similar to the native structures. AlphaFold 2 is a very useful approach to predict other membrane embedded transmembrane proteins. QTY is a useful approach for working with difficult-to-study hydrophobic proteins. The SLC transporter water-soluble QTY variants not only could be used for designs of molecular machines, but also as water-soluble antigens for generating therapeutic monoclonal antibodies and for accelerating drug discovery.

## Methods

### Protein sequence alignments and other characteristics

The native protein sequences for SLC transporters and their QTY-variant sequences are aligned using the same methods previously described^33,34^. The website Expasy (https://web.expasy.org/compute_pi/) was used to calculate the molecular weights (MW) and pI values of the proteins.

### AlphaFold2 predictions

AlphaFold2^28,30^ Program https://github.com/sokrypton/ColabFold was used for the structure predictions of the QTY variants following the instructions at the website on 2 × 20 Intel Xeon Gold 6248 cores, 384 GB RAM, and a Nvidia Volta V100 GPU. The European Bioinformatics Institute (EBI, https://alphafold.ebi.ac.uk) has all AlphaFold2-predicted structures. The Uniprot website https://www.uniprot.org has each protein ID, entry name, description, and FASTA sequence. The data was taken from UniProt using a custom Python code. The QTY method website (https://pss.sjtu.edu.cn/) can convert the FASTA protein sequences into their water-soluble versions. These steps were optimized using Python libraries for web applications such as requests and splinter.

### Superposed structures

The molecular structures are taken from PDB https://www.rcsb.org. They include SLC2A1 (PDB: 6THA)^39^, SLC7A11 (PDB: 7P9U)^40^, SLC4A4 (PDB: 6CAA)^41^, SLC1A5 (PDB: 5LMM)^42^, SLC7A5 (PDB: 6IRS)^43^, SLC29A1 (PDB: 6OB6)^44^. AlphaFold2 predictions of 8 native SLC transporters and their QTY variants were carried out using the AlphaFold2 program at https://github.com/sokrypton/ColabFold. Uniprot https://www.uniprot.org is the source for all 13 SLC transporter protein sequences and AlphaFold2 was performed to predict QTY variant structures. These structures are superposed using PyMOL https://pymol.org/2/.

### Structure visualization

Two key programs were used for structure visualization: PyMOL https://pymol.org/2/ and UCSF Chimera https://www.rbvi.ucsf.edu/chimera/. PyMOL program is used for the superposed models, while hydrophobicity models were rendered using Chimera.

### Ethical approval

(1) All methods were carried out in accordance with relevant guidelines and regulations. (2) All experimental protocols were approved by a named institutional and licensing committee. (3) Neither human biological samples, nor human subjects were used in the study. This is a completely digital structural biology study using the publicly available AlphaFold2 machine learning program.

## Data Availability

European Bioinformatics Institute (EBI, https://alphafold.ebi.ac.uk) is the deposit site for all AlphaFold2 predicted > 214 million protein structures. Please use the website for more detailed information, please contact the first author Eva Smorodin at ribes.ev@gmail.com and at website: https://github.com/eva-smorodina/slc for the QTY code designed water-soluble SLC variants.
